# Panel of polymorphic heterologous microsatellite loci to genotype critically endangered Bengal tiger: a pilot study

**DOI:** 10.1186/2193-1801-3-4

**Published:** 2014-01-03

**Authors:** Sudhanshu Mishra, Sujeet Kumar Singh, Ashok Kumar Munjal, Jouni Aspi, Surendra Prakash Goyal

**Affiliations:** Department of Animal Ecology and Conservation Biology, Wildlife Institute of India, PO Box #18, Chandrabani, Dehradun, 248001 Uttarakhand India; Department of Bioscience and Biotechnology, Banasthali University, Banasthali, 304022 Rajasthan India; Department of Zoology, Indira Gandhi National Tribal University, Amarkantak, 484886 MP India; Department of Biology, University of Oulu, PO Box 3000, 90014 Oulu, Finland

**Keywords:** Bengal tiger, *Panthera tigris tigris*, Heterologous microsatellite loci, Genotyping panel

## Abstract

In India, six landscapes and source populations that are important for long-term conservation of Bengal tigers (*Panthera tigris tigris*) have been identified. Except for a few studies, nothing is known regarding the genetic structure and extent of gene flow among most of the tiger populations across India as the majority of them are small, fragmented and isolated. Thus, individual-based relationships are required to understand the species ecology and biology for planning effective conservation and genetics-based individual identification has been widely used. But this needs screening and describing characteristics of microsatellite loci from DNA from good-quality sources so that the required number of loci can be selected and the genotyping error rate minimized. In the studies so far conducted on the Bengal tiger, a very small number of loci (n = 35) have been tested with high-quality source of DNA, and information on locus-specific characteristics is lacking. The use of such characteristics has been strongly recommended in the literature to minimize the error rate and by the International Society for Forensic Genetics (ISFG) for forensic purposes. Therefore, we describe for the first time locus-specific genetic and genotyping profile characteristics, crucial for population genetic studies, using high-quality source of DNA of the Bengal tiger. We screened 39 heterologous microsatellite loci (Sumatran tiger, domestic cat, Asiatic lion and snow leopard) in captive individuals (n = 8), of which 21 loci are being reported for the first time in the Bengal tiger, providing an additional choice for selection. The mean relatedness coefficient (R = −0.143) indicates that the selected tigers were unrelated. Thirty-four loci were polymorphic, with the number of alleles ranging from 2 to 7 per locus, and the remaining five loci were monomorphic. Based on the PIC values (> 0.500), and other characteristics, we suggest that 16 loci (3 to 7 alleles) be used for genetic and forensic study purposes. The probabilities of matching genotypes of unrelated individuals (3.692 × 10^-19^) and siblings (4.003 × 10^-6^) are within the values needed for undertaking studies in population genetics, relatedness, sociobiology and forensics.

## Background

The conservation of the tiger, among the large felids, has been a global issue because of the extinction of three sub-species (Luo et al. 2004) and the decline of 93% of the habitat of the tiger (Karanth et al. 2010). The world tiger population is reported to have declined to as low a value as 3200 (http://wwf.panda.org/what_we_do/endangered_species/tigers/tiger_resources/?188542/2010-Tiger-Factsheet) due to poaching as well as human activities that have resulted in habitat fragmentation and depletion of wild prey species across the range of the species (Sunquist et al. 1999; Linkie et al. 2006; Sanderson et al. 2006). Among the different threats to the tiger, poaching and changes in landscape patterns are the greatest (Dinerstein et al. 2007; Goodrich et al. 2008; Walston et al. 2010), and hot spots of poaching may be identified by using genetic profile data, as has been done in tracking elephant ivory (Wasser et al. 2004). Therefore, a better understanding of the species at the individual level is needed for effective conservation planning and to avoid any further extinction of the extant sub-species.

Among the extant tiger subspecies, the largest population (1706) is that of the Bengal tiger (Jhala et al. 2011), which is the national animal of India and an endangered species listed under Schedule I of the Wildlife (Protection) Act, 1972 of India. For science-based management of the species in fragmented landscapes, an understanding of its ecology, biology and genetics is crucial. The need for periodic monitoring of species genetics, especially for large carnivores occupying highly exploited and fragmented landscapes, has also been emphasized (Anderson et al. 2004). Besides, reliable methods can be used to understand the causes responsible for the changing population demography are essential for designing the Tiger Conservation Plan (TCP) (Gopal et al. 2007). However, with tigers being territorial, elusive, cryptic and nocturnal animals (Karanth et al. 2003), direct observation and enumeration are not feasible for obtaining such information.

With the recent advances in molecular ecology, non-invasive genetic sampling and microsatellite markers for multi-locus genotyping have been used in studying ecology, biology and conservation genetics (Anderson et al. 2004; Mondol et al. [Bibr CR50][Bibr CR51]; Miotto et al. 2007,2011; Charruau et al. 2011; Castilho et al. 2012; Reddy et al. 2012; Sharma et al. 2013), behavioral genetics (Langergraber and Vigilant 2011; Lyke et al. 2013) and socio-biology (Langergraber et al. 2013).

Though, microsatellites have widely been used in understanding genetics but a major constraint in the use of these loci is the need to isolate and characterize them using cloning and sequencing techniques. One of the ways of circumventing this step is to screen the variations in microsatellites developed for other related species in order to find useful loci (Moore et al. 1991; FitzSimmons et al. 1995; Shepherd et al. 2002; Mantellatto et al. 2010). Therefore, numerous attempts have been made to use heterologous primers to support the conservation genetics of felids, viz. the jaguar (*Panthera onca*) (Ruiz-Garcia et al. 2006), snow leopard (*Panthera uncia*) (Waits et al. 2007), clouded leopard (*Neofelis nebulosa*) (Wilting et al. 2007), Siberian tiger (*P. t. altaica*) (Alasaad et al. 2011), cheetah (*Acinonyx jubatus*) (Charruau et al. 2011), jaguarandi (*Puma yagouaroundi*) (Holbrook et al. 2013), Indian leopard (*Panthera pardus fusca*). (Mondol et al. 2009a; Dutta et al. 2012,2013) and Bengal tiger (Bhagavatula and Singh 2006; Mondol et al. 2009b; Reddy et al. 2012; Sharma et al. 2013). However it is also useful to have large data available through screening of microsatellite loci across species. This will provide an alternate option in selecting loci for a particular genetic study and may also lead to complement data or report if there are any discrepancies.

Most of the studies undertaken so far on the Bengal tiger (Bhagavatula and Singh 2006; Mondol et al. 2009b; Reddy et al. 2012; Sharma et al. 2013) fail to provide detailed information on locus-specific genetic characteristics (polymorphic information content [PIC] and probability of identity [P_ID_]) and genotyping profile characteristics (stutter, allele to peak height etc.). Besides, information of these studies have been from fecal DNA, except for a few loci, which have been studied using high-quality DNA (Bhagavatula and Singh 2006; Mondol et al. 2009b). Thus, selection of the best loci for use in population genetics and forensic studies and minimizing genotyping errors has hitherto been precluded.

Therefore, there is a strong need to describe locus-specific genotyping profile characteristics using DNA from a high-quality source, which has been suggested in the literature to minimize genotyping errors related to allele calling (Matsumoto et al. 2004). This has also been indicated in the guidelines of the ISFG (Gill et al. 2006,2012). Thus, we describe for the first time the screening and genotyping profile characteristics of 39 microsatellite markers developed for the Sumatran tiger (*Panthera tigris sumatrae*), domestic cat (*Felis catus*), Asiatic lion (*Panthera leo persica*) and snow leopard using DNA from a high-quality source. Of these, 21 loci are being reported for the first time in the literature for the Bengal tiger. Based on our findings, we suggest a combination of highly polymorphic dinucleotide and tetranucleotide repeat loci along with their genotyping profile characteristics for use in population genetic, forensic and non-invasive genetic sampling studies involving the Bengal tiger that will minimize allele calling errors by using locus-specific profile characteristics. Thus, the present study will provide better options in the selection and use of loci in population genetic and forensic studies carried out on Bengal tigers.

## Results and discussion

Bengal tiger DNA samples (n = 8) were amplified successfully for all 39 heterologous loci, and data analysis using MICROCHECKER 2.2.3 (Van Oosterhout et al. 2004) and GIMLET (Valiere 2002) clearly indicated the absence of null alleles, allele dropout, false alleles and scoring errors, associated with peak stuttering in genotyping data. The mean value of the relatedness coefficient (R = −0.143) also indicate that the selected tigers were not closely related to each other, as could be expected in captive individuals.

Three tetranucleotide repeat loci (Fca453, Fca731 and Fca749) and two dinucleotide repeat loci (6HDZ007 and Ple55) were found to be monomorphic in the Bengal tiger and were excluded from further analyses. In polymorphic loci (n = 34), the observed allele size ranged from 78 to 315 bp (Table 1), whereas the number of alleles (Na) per locus ranged from 2 to 7 (average 3.323). The effective number of alleles (Ne) per locus ranged from 1.438 to 4.923 (average 2.418). The average observed (H_O_) and expected heterozygosities (H_E_) for polymorphic loci were 0.625 and 0.548, respectively. Four loci (PUN82, PUN100, PUN124, Ple57) had an H_E_ level greater than 0.70. The higher value of H_O_ compared with H_E_ may be due to outbreeding that has probably taken place in a zoo as the animals were mixed from one population to another in India. A recent reduction in population size may cause a deficit of rare alleles compared with the number expected in a population at equilibrium. Since, rare alleles contribute comparatively little to H_E_, there will be an excess of H_O_ while compared with a population at equilibrium among equal number of alleles (Cornuet and Luikart 1996; Garza and Williamson 2001). This hypothesis can be used to support the relatively high H_O_ in this zoo population, in which the tigers may have different geographic origins. Hardy-Weinberg equilibrium (HWE) analysis may be affected by the small sample size, even though, we observed an HWE at all loci except 6HDZ170, F85 and Fca723. Therefore, the higher values of heterozygosity are not due to HW disequilibrium. The method of Weir and Cockerham (1984) was used to calculate the inbreeding coefficient (F_IS_), and the heterozygosity excess was examined at 68% of the loci (of which 74% and 26% were from dinucleotide and tetranucleotide repeat loci). The mean F_IS_ value of the polymorphic loci was −0.103. Fifteen pairs of loci (6HDZ089 and F41, 6HDZ089 and Fca272, F41 and Fca272, F42 and Fca304, F41 and Fca733, Fca272 and Fca733, Fca506 and Fca733, 6HDZ089 and Fca740, F41 and Fca740, F53 and Fca740, Fca272 and Fca740, Fca506 and Ple57, Fca733 and Ple57, 6HZ317 and PUN132, F41 and PUN132) indicated a significant linkage disequilibrium (LD) (P < 0.05). However, most of the selected loci of the domestic cat are located on different chromosomes. Therefore, the loci were selected carefully for the panel to avoid their physical linkage. The polymorphic microsatellite loci (n = 34) showed a mean PIC value 0.482, with sixteen loci having PIC values between 0.511 and 0.770, six loci having PIC values between 0.427 and 0.483 (which are considerably informative for population genetic studies (Botstein et al. 1980)) and the others having PIC values less than 0.400 (Table 1).

**Table 1:** **Observed size range, genetic diversity statistics and genotyping profile characteristics for 39 microsatellite loci tested on 8 captive Bengal tigers**

Locus ID	Chr. Asn.	Size range (bp)	N	Na	Ne	Ho	H_E_	PIC	F_IS_	P_ID_(locus)	P_ID_Sibs (locus)	Main allele peak (height)	Height ratio (1)	Height ratio (2)	Height ratio (3)	Height ratio (4)
6HDZ007^1^	NI	170	8	1	1.000	0.000	0.000	0	-	1.00E + 00	1.00E + 00	2000	1:20	1:03	0	0
6HDZ056^1†^	NI	172-176	8	3	2.415	0.750	0.586	0.52	−0.217	2.38E-01	5.16E-01	2700	1:03	1:03	1:1.6	1:1.4
6HDZ064^1^	NI	166-170	8	2	1.753	0.625	0.430	0.337	−0.4	4.18E-01	6.40E-01	800	1:2.6	1:08	1:6	1:1.6
6HDZ089^1^	NI	207-221	8	3	1.910	0.625	0.477	0.427	−0.25	3.23E-01	5.93E-01	500	1:05	1:12	1:1	1:02
6HDZ170^1†^	NI	216-226	8	3	2.723	0.875*	0.633	0.556	−0.324	2.12E-01	4.87E-01	6200	1:7.5	1:6.2	1:6.2	1.24
6HDZ317^1^	NI	192-206	8	2	1.882	0.750	0.469	0.359	−0.555	3.92E-01	6.14E-01	10000	1:05	1:2.5	1:5.2	1:02
6HDZ700^1^	NI	141-143	8	2	1.600	0.500	0.375	0.305	−0.272	4.61E-01	6.78E-01	800	1:2.6	1:04	1:1.6	1.1
6HDZ817^1^	NI	238-242	8	2	1.969	0.625	0.492	0.371	−0.206	3.79E-01	5.99E-01	1000	1:05	1:10	1:2.5	1:1.4
Fca008^2^	A1	130-134	8	3	2.032	0.625	0.508	0.428	−0.166	3.22E-01	5.77E-01	2300	1:03	1:1.1	1:1.8	1:1.5
Fca126^2^	B1	124-150	8	4	1.969	0.625	0.492	0.458	−0.206	2.92E-01	5.77E-01	4600	1:2.3	1:18	0	1:1.5
Fca272^2^	A3	112-122	8	3	1.684	0.500	0.406	0.371	−0.166	3.88E-01	6.44E-01	950	1:2.4	1:5.2	0	1:1.2
Fca304^2†^	A2	125-141	8	3	2.462	0.750	0.594	0.511	−0.2	2.48E-01	5.15E-01	5800	1:2	1:6.4	1:7.8	1:1.2
Fca506^2†^	F2	206-220	8	3	2.844	0.625	0.648	0.575	0.102	1.97E-01	4.75E-01	6800	1:3.4	1:4.5	1:34	1:2.2
Fca628^2†^	D2/E3	106-110	8	3	2.723	0.500	0.633	0.556	0.272	2.12E-01	4.87E-01	1900	1:19	1:12.6	1:9.5	1:1.5
Ple23^3†^	NI	152-168	8	4	2.844	0.750	0.648	0.592	−0.09	1.80E-01	4.71E-01	8000	1:04	0.097	1:16	1:1.4
Ple51^3^	NI	172-176	8	2	1.600	0.500	0.375	0.305	−0.272	4.61E-01	6.78E-01	3800	1:2.7	1:09.5	1:7.6	1:1.3
Ple55^3^	NI	148	8	1	1.000	0.000	0.000	0	-	1.00E + 00	1.00E + 00	8200	1:4.7	1:02.5	1:82	0
Ple57^3†^	NI	141-155	8	5	2.977	0.750	0.664	0.618	−0.063	1.59E-01	4.58E-01	6200	1:6.2	1:10	1:25	1:1.6
PUN82^4†^	NI	100-122	8	7	4.923	0.750	0.797	0.77	0.125	6.85E-02	3.69E-01	1690	1:2.6	1:15.6	1:5.6	1:1
PUN100^4†^	NI	78-100	8	6	3.765	0.625	0.734	0.702	0.2135	1.03E-01	4.09E-01	1030	1:4	1:17.1	0	1:1.9
PUN124^4†^	NI	88-106	8	6	4.129	0.750	0.758	0.723	0.0769	9.31E-02	3.94E-01	1140	1:3	1:09.5	1:2.1	1:1.9
PUN132^4†^	NI	117-121	8	3	2.977	1.000	0.664	0.59	−0.4545	1.87E-01	4.65E-01	2900	1:3.7	1:06.6	1:1.4	1:1.4
PUN225^4^	NI	178-184	8	3	1.471	0.375	0.320	0.294	−0.1053	4.88E-01	7.12E-01	1450	1:3.4	1:07.8	1:1.8	1:1.5
PUN229^4^	NI	106-120	8	3	2.169	0.625	0.539	0.447	−0.0938	3.05E-01	5.57E-01	1520	1:3.3	1:10.8	1:3.5	1:1.87
PUN327^4^	NI	84-90	8	2	1.882	0.250	0.469	0.359	0.5172	3.92E-01	6.14E-01	6345	1:3	1:15.9	1:5.2	1:1.5
**Mean (based only on polymorphic di-nucleotide loci, n = 23)**				3.347	2.465	0.641	0.552	0.485	−0.118							
F41^2†^	D2	170-188	8	4	2.977	0.625	0.664	0.616	0.125	1.61E-01	4.58E-01	5500	1:5	1:2.8	0	1:1.3
F42^2^	A1	207-231	8	3	1.662	0.500	0.398	0.354	−0.191	4.06E-01	6.52E-01	7000	1:23	1:14	0	1:1.5
F53^2^	A1	128-152	8	4	2.169	0.750	0.539	0.483	−0.333	2.68E-01	5.48E-01	4000	1:16	1:08	0	1.4
F85^2†^	B1	156-176	8	3	2.612	0.375*	0.617	0.544	0.447	2.20E-01	4.96E-01	1000	1:11	1:05	0	1:01
F124^2†^	E1	258-286	8	4	3.368	0.625	0.703	0.644	0.176	1.48E-01	4.35E-01	9000	1:22.5	1:18	1:09	1:1.1
Fca391^2^	B3	216-224	8	2	1.438	0.375	0.305	0.258	−0.166	5.30E-01	7.30E-01	3200	1:5.3	1:32	1:4.5	1:02
Fca441^†^	D3	148-160	8	4	2.723	0.750	0.633	0.57	−0.12	1.97E-01	4.83E-01	5500	1:11	1:27.5	1:18	1:1.3
Fca453^2^	A1	198	8	1	1.000	0.000	0.000	0	-	1.00E + 00	1.00E + 00	9000	1:9	1:09	0	0
Fca723^2^	A1	295-315	8	2	2.000	1.000*	0.500	0.375	−1	3.75E-01	5.94E-01	3000	1:15	1:06	1:7.5	1:1.5
Fca731^2^	B1	278	8	1	1.000	0.000	0.000	0	-	1.00E + 00	1.00E + 00	8200	1:41	1:08.2	1:27	0
Fca733^2^	B2	119-123	8	2	1.753	0.375	0.430	0.337	0.192	4.18E-01	6.40E-01	1300	1:8.6	1:04.3	1:2.8	1:01
Fca740^2^	C1	290-302	8	4	1.969	0.625	0.492	0.458	−0.206	2.92E-01	5.77E-01	7800	1:2.6	1:09.7	0	1:1.1
Fca742^2†^	D4	152-176	8	4	2.844	0.500	0.648	0.592	0.291	1.80E-01	4.71E-01	7100	1:7.1	1:04.7	1:7.1	1:14
Fca749^2^	F2	103	8	1	1.000	0.000	0.000	0	-	1.00E + 00	1.00E + 00	4400	1:14.6	1:04.4	1:22	0
**Mean (based on only polymorphic tetra-nucleotide loci, n = 11)**				3.272	2.319	0.590	0.539	0.475	−0.071							
**Mean (based only on overall polymorphic loci, n = 34)**				3.323	2.418	0.625	0.548	0.482	−0.103							
**Mean (based only on suggested panel of polymorphic loci, n = 16)**				4.062	3.081	0.687	0.664	0.604	0.022							

^†^Locus recommended for panel of 16 microsatellite loci.

Chr. Asn., chromosomal assignment of locus in species of origin; NI, no information; T, tetranucleotide repeat; D, dinucleotide repeat; bp, base pairs; Na, number of alleles; Ne, number of effective alleles; H_O_, observed heterozygosity; H_E_, PIC, polymorphic information content; expected heterozygosity; P_ID_ (locus), probability of identity between unrelated individuals; P_ID_ Sibs (locus), probability of identity between siblings; Height ratio 1, first stutter peak/main allele peak; Height ratio 2, minus A peak/main allele peak; Height ratio 3, plus A peak/main allele peak; Height ratio 4, heterozygote allele peak/main allele peak; PIC, polymorphic information content; F_IS,_ inbreeding coefficients_._^1^Williamson et al. (2002); ^2^Menotti-Raymond et al. (1999 2005); ^3^Singh et al. (2002); ^4^Janecka et al. (2008); *significance of Hardy-Weinberg test (*P < 0.05).

**Figure 1:**
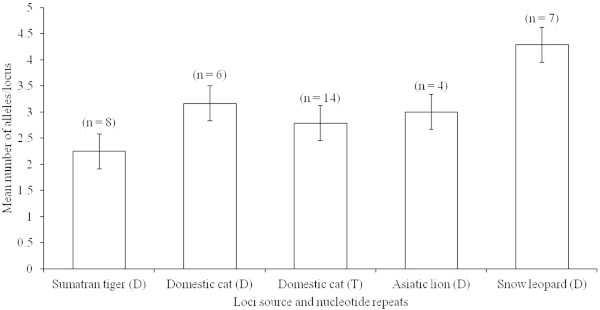
**Distribution pattern of observed mean number of alleles per locus in Bengal tigers based on the use of the dinucleotide (D) and tetranucleotide (T) repeat microsatellite loci developed for heterologous felids.** Values in parentheses are numbers (n) of loci examined.

The observed number of alleles indicates that the loci developed from the domestic cat, Asiatic lion and snow leopard have a greater number of alleles than do those from the Sumatran tiger (Figure 1). Pairwise statistical analysis (Mann–Whitney *U* test) indicates significant differences between the Sumatran tiger and domestic cat (P < 0.001), Sumatran tiger and Asiatic lion (P < 0.0001), domestic cat and snow leopard (P < 0.0001), Sumatran tiger and snow leopard (P < 0.0001 and Asiatic lion and snow leopard (P < 0.0001) but not between domestic cat and Asiatic lion (P < 0.105). This shows that the discriminatory power of the loci developed from the domestic cat, Asiatic lion and snow leopard is greater in Bengal tiger DNA samples. The majority of recent studies undertaken on felids have also used microsatellite loci developed for the domestic cat (Alasaad et al. 2011; Charruau et al. 2011; Dutta et al. 2012; Reddy et al. 2012; Holbrook et al. 2013 Lyke et al. 2013; Sharma et al. 2013). Therefore, domestic cat microsatellite loci may enable a comparison of data across species to minimize ascertainment biases (Garner et al. 2005).

The published reports indicate that there is a higher error rate for dinucleotide repeat loci than for tetranucleotide repeat loci during allele calling and this is difficult to address due to a lack of genotyping profile characteristics (Cullingham et al. 2010). Therefore, we analyzed polymorphic dinucleotide repeat loci (n = 23) and tetranucleotide repeat loci (n = 11) separately to determine the level of allelic diversity, which has a strong significant role in individual identification. The number of alleles per locus at polymorphic dinucleotide repeat loci (n = 23) ranged from 2 to 7 (average 3.347), the average observed and expected heterozygosities for these loci were 0.641 and 0.552, respectively, and the mean PIC value was 0.485 (Table 1). The number of alleles per locus at polymorphic tetranucleotide repeat loci (n = 11) ranged from 2 to 4 (average 3.272), the average observed and expected heterozygosities for these loci were 0.590 and 0.539, respectively, and the mean PIC value was 0.475 (Table 1). Our study clearly indicates that the polymorphic dinucleotide and tetranucleotide repeat loci show more or less the same genetic diversity and other characteristics. Besides, there has been a choice of using tetranucleotide over dinucleotide loci to minimize problems of allele calling (Cullingham et al. 2010). Thus, the domestic cat loci provide a better choice, with an adequate number of dinucleotide and tetranucleotide repeat loci, compared with the loci developed for the tiger and other felids so far (Figure 1; Table 1).

**Figure 2:**
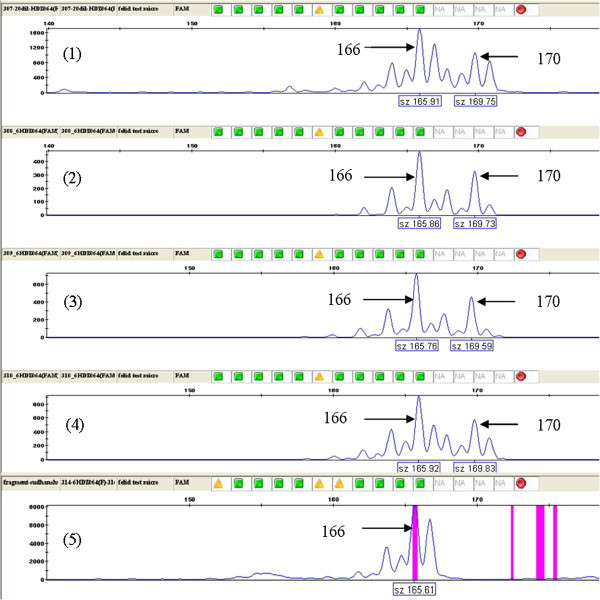
**6HDZ064 locus with 2 alleles, 166 bp and 170 bp.** All alleles show well-resolved peaks and contain stutters. Samples 1–4 are heterozygotes, and sample 5 is a homozygote.

Allele scoring was easy for all the loci analyzed, and Figure 2 shows the allele scoring of one of the loci. Matsumoto et al. (2004) emphasized a need for interpretation of the locus-specific peak patterns and characteristics and suggested a novel algorithm for automated genotyping of microsatellites. We provide information for calculating the peak ratio of the first stutter, minus A, plus A and heterozygote allele (Table 1), which will make interpretation and allele scoring by others easier and more accurate. Such information are lacking for most of the studies so far undertaken for Bengal tigers.

Hence, we suggest a panel of 16 microsatellite loci including polymorphic dinucleotide and tetranucleotide repeat loci (Table 1) for genotyping-based studies carried out to understand the genetic structure of the population and to gather information on the ecology, biology and social organization of the Bengal tiger from skin, tissue, fecal and hair samples. The suggested panel of 16 loci has 3 to 7 alleles per locus (average 4.062); the average observed and expected heterozygosities for these loci were 0.687 and 0.664, respectively; and the mean PIC value was 0.604 (0.511–0.770). Only two pairs of loci (F41 and PUN132, Fca506 and Ple57) showed a significant LD (P < 0.05), while chromosome location of PUN132 and Ple57 is not known (Table 1). Therefore, it should be checked whether they are also linked in other Bengal tiger populations. The mean F_IS_ value of the suggested panel was also close to zero (0.022), which indicates that the selected captive population of Bengal tigers (n = 8) is in HWE.

**Figure 3:**
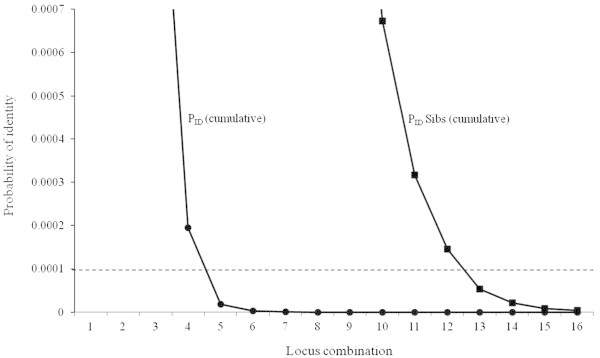
**Probability of identity of unrelated individuals (P**_**ID**_ **) and probability of identity of siblings (P**_**ID**_ **sibs) in locus combination using selected panel (n = 16).**

The probability of identity (P_ID_), or probability of having the same genotype at multiple microsatellite loci of two individuals if they are drawn at random from a population, can be valuable information in a study where individual identification is needed. It can be estimated for differing number of loci (Waits et al. 2001). A P_ID_ value of <0.01 (1 in 100) is considered essential for genetic studies in which population size estimation is required (Mills et al. 2000). However, a sufficiently low P_ID_ value of 0.001–0.0001 has been recommended in wildlife forensic applications for law enforcement (Waits et al. 2001; Eiken et al. 2009; Lorenzini et al. 2011). A P_ID_ level of <0.0001 has been used to study the population genetics of the bear and wolf (Waits et al. 2001). Figure 3 indicates that a combination of 5 polymorphic microsatellite loci from recommended panel (n = 16) was necessary to reach a P_ID_ level of <0.0001 to adequately discriminate between individual tigers but was not sufficient for identification of siblings (P_ID_ > 0.02). However, a combination of 12–16 selected polymorphic heterologous microsatellite loci (Table 1) was adequate to reach a P_ID_ level of <0.0001 for discriminating siblings. The probability of identity of unrelated individuals determined using 16 polymorphic heterologous microsatellite loci was P_ID (cumulative)_ = 3.692 × 10^-19^ and of siblings P_ID_ Sibs (cumulative) _=_ 4.003 × 10^-6^, and thus it even meets the requirements of forensic studies, as suggested by Waits et al. (2001). The reported numbers of individuals in tiger populations in different protected areas of India range from 4 to 718 (Jhala et al. 2008), and some of the populations may be considered to be highly inbred due to isolation and small population sizes. We recommend the use of the suggested panel of 16 loci (Table 1) as it will not lead to any misidentification between two individuals, including siblings, in small or inbred Bengal tiger populations. At the same time, a larger number of loci may introduce more genotyping errors when a low-quality source of DNA (viz. scat) is used (Creel et al. 2003). But the multiple-tube approach (Navidi et al. 1992; Goossens et al. 1998) and two-step multiplex PCR method can be used to overcome this problem without compromising the number of loci (Arandjelovic et al. 2009; Chang et al. 2012), which are crucial for use in studies related to the ecology and biology of a species.

When using different loci in studies involving samples that have been obtained non-invasively, the researcher is keen to know the error rates and amplification success rate. We tested the applicability of the recommended panel with noninvasive samples (scat) and blood from the same individuals and estimated the frequency of occurrence of genotyping error rates. The values of the mean genotyping error rates were low and considerable for non-invasive genetic studies (allele dropout, 0.004 ± 0.002 SD; false allele, 0.004 ± 0.002 SD and scoring error, 0.006 ± 0.003 SD). These relatively low error rates may be due to the use of locus-specific profile characteristics, which leads to correct decisions in allele calling. We also did not observe any change or discrepancy in the genetic data compared with the data generated from blood samples.

The key issue when using non-invasive genetic samples, which are normally from poor-quality sources of DNA (especially scats), is identification and selection of loci that should have a higher amplification success rate as errors related to genotyping may be addressed by using other approaches that have been suggested (Matsumoto et al. 2004; Cullingham et al. 2010). We further tested our suggested panel of 16 markers and validated it with 50 scat samples collected from different Bengal tiger populations in India (Mishra et al. 2012). The preliminary results indicate that the average amplification success rate is 66% with field-collected scat samples tested with a selected panel of 16 loci (Mishra et al. 2012), compared with other studies on carnivores, in which the reported success with fecal DNA is between 53% and 75% (Bellemain and Taberlet 2004; Bellemain et al. 2005; Smith et al. 2006; Murphy et al. 2007; Hansen et al. 2008).

Our results of heterologous microsatellite loci, which have already been used in other studies, and additional loci (n = 21) will provide a wider choice for future efforts to assess the genetic diversity, existing range and genetic assignment of different populations of free-ranging Bengal tigers and minimize errors in allele calling.

## Material and methods

### Sample collection

The first step before applying the non-invasive genotyping method to population monitoring and other aspects of the ecology and biology of the Bengal tiger is to identify a suite of hypervariable microsatellite loci using known good-quality tiger samples. To accomplish this, we obtained blood samples of 8 captive Bengal tigers which were sent to Wildlife Institute of India, Dehradun, India from Mahendra Chaudhury Zoological Park, Chhatbir, Mohali, India for DNA profiling. The histories of individual tigers and their translocation are inadequately documented in the *Indian National Studbook for Bengal Tigers*, *2011*. Therefore, the place or geographic origin of these individuals is unknown. The reason behind opting for these individuals in the present study is that if any microsatellite locus shows polymorphism in a captive population, that locus is supposed to show more polymorphism with wild individuals, which are thought to be outbred. DNA was extracted from their blood samples using Bio Robot EZ1 (Qiagen, Germany).

Scat samples from the same captive individuals (n = 8) and 50 scat samples from wild tigers were collected. A QIAamp DNA Stool Mini Kit (Qiagen, Germany) was used, following the manufacturer’s protocol, to extract DNA from the scat samples.

### Selection, screening and genotyping of DNA from blood samples using heterologous microsatellite loci

We selected and screened 25 dinucleotide and 14 tetranucleotide microsatellite loci that have been developed for the Sumatran tiger (*Panthera tigris sumatrae*) (Williamson et al. 2002), Asiatic lion (Singh et al. 2002), domestic cat (Menotti-Raymond et al. 1999 and 2005) and snow leopard (Janecka et al. 2008) to examine their allelic size range and polymorphism level in the Bengal tiger (Table 2). Polymerase chain reactions (PCR) were carried out in an Applied Biosystems 9700 thermocycler (Applied Biosystems, Germany) in a 10 μl reaction mixture containing 1 × PCR ABI Taq gold buffer, 2.0 mM MgCl_2_, 0.4 mM dNTP mix, approximately 50 ng genomic DNA, 4 pmol forward and reverse primers and 1 U Taq Gold DNA Polymerase (Applied Biosystems). Amplification was attempted for all 39 loci for all samples using PCR amplification conditions that have been published in the literature (Williamson et al. 2002; Singh et al. 2002; Menotti-Raymond et al. 1999,2005; Janecka et al. 2008). The amplified PCR products were checked on 2% agarose gel in a 1 × TAE buffer.

**Table 2:** **Number of microsatellite loci, including dinucleotide and tetranucleotide repeats, from different species screened with Bengal tiger DNA samples**

S. No.	Loci (n)	Loci repeats		References
		Dinucleotide	Tetranucleotide	
Sumatran tiger	8	8	-	Williamson et al. (2002)
Asiatic lion	4	4	-	Singh et al. (2002)
Domestic cat	20	6	14	Menotti-Raymond et al. (1999 2005)
Snow leopard	7	7	-	Janecka et al. (2008)

### Statistical analyses

The PCR products were scored on an ABI 3130 fluorescence detection system using the GeneMapper software package (Applied Biosystems). The quality of the microsatellite data was evaluated statistically for errors in genotyping arising from null alleles (non-amplified alleles). Stutter peaks were scored using Micro-Checker 2.2.3 (Van Oosterhout et al. 2004). The frequencies of occurrence of large-allele dropout (short-allele dominance) and false allele were computed using GIMLET (Valiere 2002). To ascertain and obtain reliable genotypes, DNA from all eight blood, eight scats of captive Bengal tigers and fifty field collected scat samples were re-genotyped three to four times, respectively, at all the microsatellite loci screened so far (n = 39). Genetic diversity statistics for number of alleles (Na), number of effective alleles (Ne), observed heterozygosity (H_O_) and expected heterozygosity (H_E_) were generated using GenAlEx 6 (Peakall and Smouse 2006) and genepop’007 (Rousset 2008). Using the allele frequencies, the polymorphic information content (PIC) of the markers was calculated using Cervus (ver. 3.0) (Kalinowski et al. 2007). The expected probability of matching genotypes for unrelated individuals (P_ID_) and siblings (P_ID_ Sibs) was calculated for each locus using GIMLET (Valiere 2002). genepop’007 (Rousset 2008) was used to test the deviation from HWE. The F_IS_ was determined using the probability test approach (Guo and Thompson 1992), with 10,000 dememorizations, 500 batches and 10,000 iterations per batch in genepop’007 (Rousset 2008). The inbreeding coefficients and the linkage disequilibrium (LD) were also tested using genepop’007 (Rousset 2008). Considering the lack of details regarding individual tigers in the *Indian National Studbook for Bengal Tigers*, *2011*, we estimated the Queller and Goodnight relatedness coefficients (Queller and Goodnight 1989) using GenAlEx 6 (Peakall and Smouse 2006). To ensure that the selected individuals were not related to each other, the level of relationship among the individuals was established using the R-value as suggested by Blouin (2003) and was calculated using GenAlEx 6 (Peakall and Smouse 2006).
